# Systematic review of educational interventions for looked‐after children and young people: Recommendations for intervention development and evaluation

**DOI:** 10.1002/berj.3252

**Published:** 2016-11-17

**Authors:** Rhiannon Evans, Rachel Brown, Gwyther Rees, Philip Smith

**Affiliations:** ^1^ DECIPHerCardiff UniversityUK; ^2^ WISERDCardiff UniversityUK; ^3^ CASCADECardiff UniversityUK

**Keywords:** foster care, group home, intervention, systematic review

## Abstract

Looked‐after children and young people (LACYP) are educationally disadvantaged compared to the general population. A systematic review was conducted of randomised controlled trials evaluating interventions aimed at LACYP aged ≤18 years. Restrictions were not placed on delivery setting or delivery agent. Intervention outcomes were: academic skills; academic achievement and grade completion; special education status; homework completion; school attendance, suspension, and drop‐out; number of school placements; teacher‐student relationships; school behaviour; and academic attitudes. Fifteen studies reporting on 12 interventions met the inclusion criteria. Nine interventions demonstrated tentative impacts. However, evidence of effectiveness could not be ascertained due to variable methodological quality, as appraised by the Cochrane risk of bias tool. Theoretical and methodological recommendations are provided to enhance the development and evaluation of educational interventions.

## Introduction

Looked‐after children and young people (LACYP) are educationally disadvantaged compared to the general population (Sebba *et al*., 2015). They are less likely to complete primary or secondary education (Berlin *et al*., 2011; Vinnerljung & Hjern, 2011; Sebba *et al*., 2015), and academic attainment is systematically lower (Vinnerljung & Hjern, 2011; Berger *et al*., 2015). Higher education is impacted, with those in foster care being half as likely to have a university degree or equivalent by age 26 (Vinnerljung & Hjern, 2011). There is also a report of an excess risk in regard to school absence and exclusion (Sebba *et al*., 2015), with care‐experienced women being more than three times as likely to be permanently excluded (Viner & Taylor, 2005). A range of negative‐life course events likely influenced by educational disadvantage are also more prevalent in individuals who have been in care, including unemployment, homelessness and receipt of social welfare (Viner & Taylor, 2005; Berlin *et al*., 2011).

There remains limited theoretical and empirical consideration of the reasons why LACYP have poorer educational outcomes, although some explanations have been posited. These include: limited and variable access to the educational system (Zetlin *et al*., 2006); home and school placement instability (Pecora, 2012; Sebba *et al*., 2015); weak family and social networks (Franzen & Vinnerljung, 2006; Berlin *et al*., 2011); and insufficient accountability or monitoring of academic outcomes by the care system (Zetlin *et al*., 2006). However, extensive debate abounds about the risk of a selection effect, which may give rise to underlying explanatory confounders (O'Higgins *et al*., 2015). As Stone (2007) attests, there are a range of non‐random factors, including maltreatment, socioeconomic deprivation, race and ethnicity that predict entry into care but may also independently explain educational disadvantage.

### Effective educational interventions for children and young people in the care of the state: A limited evidence base

Despite emerging evidence to suggest the relative educational deficits experienced by LACYP, there remains a dearth of effective interventions directly targeted at this population or addressing them as a key subgroup within universal approaches. Forsman and Vinnerljung (2012) published a scoping review, restricting study inclusion to randomised controlled trials (RCTs), quasi‐experiments and pre‐post testing. They identified only 11 evaluations, drawing tentative conclusions of effectiveness and suggesting that interventions targeting this population may have a chance of generating positive results. However, a number of limitations were identified.

First, there remains an insufficiency of theoretically driven interventions, and where theoretical approaches have gained traction, as with the Early Start to Emancipation Preparation (ESTEP) Tutoring programme, interventions have been ineffective (Courtney *et al*., 2008; Zinn & Courtney, 2014). Hence based on existing research, it is not only premature to suggest that educational outcomes are amenable to intervention, but it is impossible to draw conclusions about the types of interventions that might work best.

Second, reported intervention impacts tend to be the product of discrete and isolated incidents of evaluation, with limited examples of replication. The only exception is Teach Your Children Well, which has been evaluated as both an individualised (Flynn *et al*., 2011; 2012; Marquis, 2013) and group‐based approach (Harper, 2012; Harper & Schmidt, 2012). Within a context where evaluation science is increasingly driven by the dictum of ‘What works for whom in what circumstances’ (Pawson & Tilley, 1997) rather than the simple question of what works, it is vital to address the extent to which interventions can reproduce the impacts demonstrated in the original trial and the degree to which adaptations may be required to accommodate cultural specificities.

Third, and perhaps most fundamentally, existing evidence of effect is compromised by a lack of scientific rigour, with limited utilisation of RCTs, which arguably constitute the most appropriate methodology to answer questions of effectiveness (Mezey *et al*., 2015). Studies have been further undermined by small sample sizes, with many failing to provide sample size calculations, making it unclear whether evaluations are sufficiently powered to detect anticipated effect sizes. Taken together, these limitations suggest the need to continually monitor and appraise the development and evaluation of educational interventions for this population, and to identify examples of best practice where possible, which may serve as a template for future methodologically robust studies.

### Review aims

The present systematic review has two aims:

1. Systematically synthesise the evidence of the effectiveness of interventions addressing the educational outcomes of LACYP, as evaluated by use of an RCT. Evaluations were limited to the utilisation of RCTs as this study design generates the most scientifically robust evidence of effect.
2. Appraise the quality of RCTs evaluating interventions addressing the educational outcomes of LACYP, in order to identify methodological limitations, recognise exemplars of best practice, and develop recommendations for future conduct and reporting.

## Methodology

The review was conducted in adherence with the PRISMA statement for the reporting of systematic reviews (Shamseer *et al*., 2015).

### Inclusion criteria

Studies were identified from 1989, to coincide with the inception of the Children's Act in the UK (1989), which allocates statutory obligations to ensure the safeguarding of children. Randomised controlled trials (RCTs) were identified for inclusion. Study participants comprised children and young people aged 18 years or younger who were currently looked‐after or had previous care experience. All delivery settings, delivery agents, intervention composition and duration were relevant. No restrictions were placed on the comparator group. Studies were required to report on a range of educational outcomes: academic skills; academic achievement and grade completion; special education status; homework completion; school attendance, suspension and drop‐out; number of school placements; teacher‐student relationships; school behaviour; and academic attitudes.

### Search strategy and information sources

A search strategy was developed in Ovid MEDLINE before being adapted to the search functions of each database. MEDLINE was used to refine the strategy as a number of interventions had a primary focus on developmental and cognitive processes, and we wanted to sensitise the search to capture these publications. Twelve relevant electronic bibliographic databases were searched in December 2014 through March 2015. Searches were limited to the English language. Searches were conducted in: ASSIA (Proquest); British Education Index (Ebsco); CINAHL (Ebsco); Education Resources Information Center (Ebsco); Embase (OVID); Medline (OVID); Medline in Process (OVID); Social Care Online; Social Science Citation Index (Web of Science); Social Services Abstracts (Proquest); Scopus (Elsevier); PsycINFO (OVID). A panel of international experts were contacted for recommendation of relevant published and unpublished evaluations. Reference lists of included studies were scanned to identify additional studies. Reviewers of the original study manuscript suggested studies for consideration.

### Study selection

Retrieved studies were exported into Endnote and duplicates were removed. Two review authors independently screened study titles and abstracts against the inclusion criteria. A third reviewer adjudicated discrepancies in decisions. Two review authors independently screened the full‐text of studies. Disagreement was resolved through discussion.

### Data extraction, data items and summary measures

The Cochrane data extraction and appraisal form was adapted to generate a standardised pro‐forma for extraction. Two reviewers extracted data. Abstracted items included: intervention group demographics; control group demographics; intervention setting and design; study design; outcome measurements; methods of analysis; process evaluation data; economic evaluation data; intervention effects. Educational summary measures were reported in the following domains: academic skills; academic achievement and grade completion; special education status; homework completion; school attendance, suspension and drop‐out; number of school placements; teacher‐student relationships; school behaviour; and academic attitudes. Measurements were not pre‐specified by the review.

### Risk of bias

The Cochrane collaboration tool for assessing the risk of bias in randomised controlled trials was employed to appraise studies (Higgins & Green, 2011). Two review authors assessed risk of bias. Domains assessed were: sequence generation; allocation concealment; blinding; completeness of data; and selective outcome reporting. Each domain was determined to be of a low or high risk of bias. Where there was insufficient detail a domain was judged as unclear. Additional risks of biases, such as confounding, were also documented.

### Synthesis of results

Studies were insufficiently homogenous so meta‐analysis could not be conducted and the review is presented narratively.

## Results

Searching of electronic bibliographic databases retrieved 2,514 studies. Consultation with experts identified 16 studies. Scanning of reference lists elicited an additional three studies. After the removal of duplicates 1,620 studies remained. Study titles and abstracts were assessed. A further 1,560 were excluded, leaving the full texts of 60 studies. Forty‐six papers did not meet the inclusion criteria, for the following reasons: children and young people in care were not the study focus, either as the primary population or a subgroup (n = 11); the intervention did not include educational outcomes (n = 29); evaluation did not include an RCT (n = 5); one study could not be located. One RCT, the Letterbox Club, which was in progress during preparation of the original manuscript reported findings during the review process and was subsequently included. Fifteen studies, reporting on 12 educational interventions, were included in the review.

### Intervention setting, delivery agent, timing and duration

One intervention was delivered within the school setting. Kids in Transition to School is a classroom‐based programme delivered 2 months prior to kindergarten entry and during the first 2 months of school (Pears *et al*., 2013). Children attend 24 sessions that address early literacy skills, prosocial skills and self‐regulatory activities. Sessions comprise 12–15 children and are delivered for a period of 2 hours, twice weekly in the first phase, and once weekly in the second phase. Carers attend eight parallel meetings intended to develop their capacity to support the child in practicing new skills, routines and behaviour. Groups are delivered for 2 hours every two weeks. Child sessions are delivered by a graduate‐level teacher and two assistant teachers, and the carer group is delivered by a facilitator and assistant, with all completing a standardised 40‐hour training programme. Participants also receive supplemental materials to support the implementation of new skills.

One intervention was delivered in the care setting, where undergraduate and graduate students are the delivery agents. In the ESTEP programme young people meet with a tutor twice a week within the care setting, and receive up to 50 hours of tutoring in a math, spelling, reading and vocabulary curriculum (Courtney *et al*., 2008; Zinn & Courtney, 2014). Tutors receive one day of training on commencement of the intervention and ongoing development twice a year. A mentoring relationship is also anticipated, with youth acquiring the skills and experience to develop healthy relationships.

Five interventions were delivered by carers within the care setting. Three were versions of the Teach Your Children Well (TYCW) approach (Flynn *et al*., 2011; 2012; Harper, 2012; Harper & Schmidt, 2012; Marquis, 2013), and two were focused on Multidimensional Treatment Foster Care (Leve & Chamberlain, 2007; Green *et al*., 2014). The individual‐level Teach Your Children Well focuses on direct one‐to‐one instruction by trained foster carers (Flynn *et al*., 2011; 2012; Marquis, 2013). Intervention includes 3 hours of instruction per week, comprising 2 hours one‐to‐one instruction in reading, 30 minutes reading aloud by the foster child, and 30 minutes self‐paced instruction in maths. The small‐group‐based Teach Your Children Well involves one or two trained university students delivering the curriculum to 3–4 children (Harper, 2012; Harper & Schmidt, 2012). Although both studies report on the same evaluation, the duration on TYCW in Harper and Schmidt (2012) is 25 weeks and 30 weeks in Harper (2012). Multidimensional Treatment Foster Care for Adolescents (MTFC‐A) delivers training and supervision to specialist foster parents for a nine‐month period, with a short period of aftercare (Green *et al*., 2014). The Multidimensional Treatment Foster Care (MTFC) intervention reported by Leve and Chamberlain (2007) caters to young girls leaving the juvenile system. The intervention involves movement into a specialist foster placement for an average duration of 174 days.

One intervention was delivered in the care setting, but there was no delivery agent and the learning was child‐led. The Letterbox Club is a gifting intervention that provides personalised educational resources to children in foster care, with resources including books, stationary items and mathematical games (Mooney *et al*., 2016). Parcels are delivered to the child on a monthly basis over a six‐month period. The intervention does not rely on, expect or demand foster carer involvement.

Three interventions were non‐standardised in their setting, delivery agent and duration. Head Start is a holistic, wraparound set of services intended to support disadvantaged pre‐school‐age children. As the largest publicly financed early education and care program in the United States, it has been subjected to numerous evaluations, but Lipscomb *et al*. (2013) provide the first evidence of effect on children in non‐parental care. The Fostering Individualized Assistance Program (FIAP) is delivered by family specialists who serve as family‐centred, clinical case managers and home‐based counsellors working across all settings in tailoring services for individual children (Clark *et al*., 1998). Zetlin *et al*. (2004) report on the effect of introducing education specialists. As a certified special education teacher, with knowledge of the rules and regulations of the school system and resources in the local community, the specialist receives referrals from child‐welfare agencies when social workers are unable to resolve educational difficulties. On receipt of a referral, the specialist advises the welfare agency, advocates for the young person, and investigates alternative school options.

One intervention was delivered to young people who had left residential care. On The Way Home (Trout *et al*., 2013) is a 12‐month intervention to support the transition of youth with or at risk of disabilities as they reintegrate into home following a stay in out‐of‐home care. Each family is assigned a trained family consultant who delivers the majority of the intervention. The programme integrates three interventions: Check & Connect, which entails the consultant working with a school mentor to monitor school engagement and communicating with the youth and parents to ensure engagement in educational goals; Common Sense Parenting, which is a series of six one‐to‐one sessions to educate parents in the skills required to support academic and behavioural success; and homework support. Over the duration of the intervention family consultants spend approximately 138 hours with each family.

### Study power

To detect the intended effect of an intervention a power calculation is required to determine the appropriate sample size. Both the evaluation of the individual‐level Teach Your Children Well (Flynn *et al*., 2011; 2012; Marquis, 2013) and the 30‐week group‐based Teach Your Children Well (Harper, 2012) were sufficiently powered. However, Green *et al*. (2014) calculated a target sample of 130 participants in order for the RCT to yield an 80% chance of finding a significant difference, but only a sample of 34 was achieved. Evaluation of the Letterbox Club was sufficiently powered to detect a minimum effect size of d = 0.47 (alpha = 0.05, estimated adjusted R^2^ = 0.60), but based on an anticipated effect size of between 0.20 and 0.30 the trial was underpowered (Mooney *et al*., 2016). The remaining nine studies did not report a power calculation.

### Risk of bias

The level of risk across the 15 studies is presented in Table 1.

**Table 1:** Risk of bias assessment

	Random sequence generation	Allocation concealment	Blinding of participants or personnel	Blinding of outcome assessment	Incomplete outcome data	Selective outcome reporting
Clark *et al*. (1998)	Low	Unclear	High	Unclear	Low	Unclear
Courtney *et al*. (2008); Zinn & Courtney (2014)	Unclear	Unclear	High	Unclear	High	Unclear
Flynn *et al*. (2011); Flynn *et al*. (2012); Marquis (2013)	Low	Unclear	High	Unclear	High	Unclear
Green *et al*. (2014)	Low	Low	High	Low	High	Unclear
Harper (2012)	Low	Unclear	High	Unclear	Low	Unclear
Harper & Schmidt (2012)	Low	Unclear	High	Unclear	Low	Unclear
Leve & Chamberlain (2007)	Unclear	Unclear	High	Unclear	Low	Unclear
Lipscomb *et al*. (2013)	Unclear	Unclear	High	Unclear	Unclear	Unclear
Mooney *et al*. (2016)	Low	Unclear	High	Unclear	Low	Unclear
Pears *et al*. (2013)	Unclear	Unclear	High	Low	Low	Unclear
Trout *et al*. (2013)	Unclear	Unclear	High	Unclear	Low	Unclear
Zetlin *et al*. (2004)	Unclear	Unclear	High	Unclear	High	Unclear

#### Random sequence generation

Seven studies did not report use of random sequence generation in the randomisation process. Eight studies stipulated using randomiser programmes.

#### Allocation concealment

Fourteen studies did not report on allocation concealment. Green *et al*. (2014) randomised according to a predefined randomisation schema, with the process being independently carried out by a different statistical group.

#### Blinding of participants or personnel

In social interventions blinding is often unfeasible. Thus although studies were unclear about how much knowledge individuals had of their trial status we can assume a level of risk.

#### Blinding of outcome assessment

Thirteen studies were unclear if evaluators where blinded when assessing outcomes. Two studies had a lower level of risk. Green *et al*. (2014) state that outcome measures were coded and masked to group allocation, with data being pooled and triangulated across reports, records and telephone interviews in order to minimise reporting bias. Pears *et al*. (2013) report that all data collection staff were blind to the group assignment of both children and caregivers.

#### Incomplete outcome data

Seven studies were judged to have low risk of bias with more than 80% retention at follow‐up. Where data were missing on one or more outcome variables analysis had often been employed to provide unbiased estimates. Seven studies were judged to have a high risk of bias either due to a retention rate of less than 80% at follow‐up, an imbalance of incomplete data across intervention and control groups, or failure to generate unbiased estimates of missing data in analysis. One study was unclear about the completeness of outcome data.

#### Selective outcome reporting

No studies stipulated that a protocol was published in advance of the review, and no protocols could be located. It is, therefore, unclear if all outcomes are reported on.

#### Confounding

Although RCTs should prevent the issue of confounding, as the intervention constitutes the only significant difference between the intervention and the control group, reported baseline differences combined with inadequate randomisation procedures, ensured that it remained a risk. Only four studies controlled for a range of covariates in analysis (Leve & Chamberlain, 2007; Lipscomb *et al*., 2013; Pears *et al*., 2013; Trout *et al*., 2013). Seven studies controlled for baseline scores of the outcome measurement (Flynn *et al*., 2011; 2012; Harper, 2012; Harper & Schmidt, 2012; Marquis, 2013; Green *et al*., 2014; Mooney *et al*., 2016). Four studies did not report controlling for covariates (Clark *et al*., 1998; Zetlin *et al*., 2004; Courtney *et al*., 2008; Zinn & Courtney, 2014).

#### Contamination

Although contamination was not explored across all studies, the transience of the sample and limited awareness of the trial status of young people by delivery agents ensured that it was a risk. In the evaluation of the ESTEP programme 12.3% of the control group received the intervention (Courtney *et al*., 2008; Zinn & Courtney, 2014). Furthermore, 18.9% of the controls received school‐based tutoring from a non‐ESTEP provider during the trial. Contamination undermined the intended intention to treat analysis.

### Outcomes of intervention evaluations

Educational outcomes of interventions are presented in Table 2.

**Table 2:** Randomised controlled trials of educational interventions

Study	Country	Intervention	Intervention group	Comparator group	Post‐baseline report	Outcomes	Covariates
Clark *et al*. (1998)	USA	Fostering Individualized Assistance Program (FIAP)	Foster care 7–15 yrs n = 54	Foster group home; Emergency shelter group home; Detention/private child‐care facility 7–15 yrs n = 77	42 mths	Extreme school absences (>40% school days missed): ns; School dropout (>1% school days): ns; Days on suspension: *OR = *2.5, *p *<* *.05*;* Extreme number of school‐to‐school movements (>3/year): ns	Not reported
Courtney *et al*. (2008); Zinn & Courtney (2014)	USA	Early Start to Emancipation Preparation (ESTEP)	Foster care; Kinship care; Group home; Other residential care. 14–15 yrs n = 246	Foster care; Kinship care; Group home; Other residential care 14–15 yrs n = 219	Avg. 26.8 mths	Letter word identification: *E.S. = *0.10; Calculation: *E.S. = *−0.01; Passage comprehension: *E.S = *−0.01; Grade level completed: *E.S. = *−0.03; GPA: *E.S. = *0.03; High school diploma or GED: *E.S. = *−0.01; School behaviour: *E.S. = *−0.05	Not reported
Flynn *et al*. (2011); Flynn *et al*. (2012); Marquis (2013)	Canada	Teach Your Children Well (TYCW)	Foster care 6–13 yrs n = 42	Foster care 6–13 yrs n = 35	30 wks	Word reading: *E.S. = *0.19, *p = *.19; Sentence comprehension: *E.S. = *0.38, *p = *.035; Spelling: *E.S. = *−0.08, *p = *.37; Math Computation: *E.S. = *.46, *p = *.009	Baseline scores
Green *et al*. (2014)	UK	Multidimensional Treatment Foster Care (MTFC‐A)	Foster care; 10–17 yrs n = 20	Foster care; Residential care; Secure unit 10–17 yrs n = 14	12 mths	Scholastic/language skills: *OR = *0.6 (*95% CI = *0.15,2.4) School attendance: *OR = *2.5, (*95% CI *= 0.48,13.1)	Baseline scores
Harper (2012)	Canada	Teach Your Children Well (TYCW) (30 weeks)	Foster care; Kinship care; 6–13 yrs n = 51	Not reported 6–13 yrs n = 50	30 wks	Word reading: *E.S. *= 0.40; Sentence comprehension: *E.S. *= 0.15, *p *= ns; Spelling: *E.S. = *0.25, *p *=* *.02; Math computation *E.S. *= 0.34, *p = *.044	Baseline scores
Harper & Schmidt (2012)	Canada	Teach Your Children Well (TYCW) (25 weeks)	Foster care; Kinship care; 6–13 yrs n = 33	Not reported 6–13 yrs n = 35	25 wks	Word reading: *E.S. *= 0.42, *p *=* *.002; Sentence comprehension: *E.S. = *0.095, *p *= ns; Spelling: *E.S. = *0.38, *p *=* *.004; Math computation *E.S. = 0.26, p *= ns	Baseline scores
Leve & Chamberlain (2007)	USA	Multidimensional Treatment Foster Care (MTFC)	Girls within the juvenile justice system; 13–17 yrs n = 37	Group care; 13–17 yrs n = 44	3–6 mths; 12 mths	Homework completion 3‐6 months: *p < *.05; Homework completion 12 month: *p *<* *.05; School attendance 12 month: *p *<* *.05	Baseline scores;
Lipscomb *et al*. (2013)	USA	Head Start	Non‐parental care 3–4 yrs n = 154	Non‐parental care 3–4 yrs n = 99	6 mths; 18 mths	6 months: Pre‐academic skills: *ß. *= 0.16, *p = *.02; Student‐teacher relationship: *ß. = *0.30, *p *<* *.01; 18 months indirect effects: Pre‐academic skills: *ß. *= 0.12, *p = *.05; Student‐teacher relationship: *ß. = *0.17, *p = *.02	Sex; Age; Special needs; Reading; Household income; Carer style; Carer change
Mooney *et al*. (2016)	UK	Letterbox Club	Foster care 7–11 yrs n = 56	Foster care 7–11 yrs n = 60	Avg. 8 months	Reading accuracy: E.S. = 0.068 *(95% CI = *−0.296,0.432*)* Reading comprehension: E.S. = −0.031 (*95% CI *= −0.395,0.333) Reading rate: E.S. = −0.233 (*95% CI = *−0.598,0.133) Recreational reading: E.S. = −0.115 (*95% CI = *−0.480,0.249) Academic reading: E.S. = −0.096 (*95% CI = *−0.460,0.268) Like school: E.S. = −0.198 (*95% CI = *−0.564,0.168) Reading accuracy: E.S. = −0.056 (*95% CI = *−0.420,0.308)	Baseline scores;
Pears *et al*. (2013)	USA	Kids in Transition to School	Foster care ≤6 yrs n = 102	Foster care ≤6 yrs n = 90	2 mths	Early literacy skills: *E.S. *= 0.26	Gender; IQ; Type of foster care; Ethnicity Prior early childhood education
Trout *et al*. (2013)	USA	On the Way Home (OTWH)	Young people with/ risk of disabilities leaving residential care 13–18 yrs n = 47	Young people with/ risk of disabilities leaving residential care 13–18 yrs n = 41	3 mths; 6 mths; 9 mths; 12 mths	Maintaining enrolment in school setting: *OR *= 0.30 (*95% CI = *0.12,0.75)	Not reported
Zetlin *et al*. (2004)	USA	Education Specialist	Foster care 5–17 yrs n = 60	Foster care 5–16 yrs n = 60	24 mths	Maths test achievement scores: *p = *.082; Reading test achievement scores: *p = *.448*;* GPA: *p *= ns; Daily attendance: *p *<* *.03; Special education status: *not reported* Number of schools attended: *p *<* *.05	Not reported

#### Academic skills

Academic skills, which predominantly constitute reading and mathematical computation, were assessed in nine interventions across twelve studies. Two validated measures were routinely employed, with five utilising the Wide Range Achievement Test (WRAT‐4) and three implementing the Woodcock Johnson Tests of Achievement III. Pears *et al*. (2013) used the Dynamic Indicators of Basic Early Literacy Skills (DINELS), Concepts About Print Test, and caregiver ratings. Green *et al*. (2014) utilised the Health of the Nation Outcome Scales for Children and Adolescents (HoNOSCA). Mooney *et al*. (2016) employed the Neale Analysis of Reading Ability test (Neale, 1997). Zetlin *et al*. (2004) did not report the measure construct.

Seven studies reporting on five interventions found some evidence of effectiveness. Kids in Transition to School measured early literacy skills in children aged six and under, finding a small effect of 0.26 (Pears *et al*., 2013). Head Start also found an effect at 6 months post‐baseline (*ß = *0.16, *p = *0.02) which was reported as significant. At 18 months post‐baseline there was no significant direct intervention effect, but there was a modest indirect effect, with gains in pre‐academic skills, establishment of positive teacher‐child relationships, and change in behaviour problems during Head Start predicting higher pre‐academic skills in the following year (Lipscomb *et al*., 2013).

The individual‐level Teach Your Children Well (Flynn *et al*., 2011; 2012; Marquis, 2013) reported positive effects on sentence comprehension (*E.S. *= 0.38*, p = *.035) and math computation (*E.S. = *0.46*, p = *.009). There was no significant impact on word reading or spelling. To note, Flynn *et al*. (2011; 2012) report Hedges g, which have been included in this review rather than the Cohen's d presented in Marquis (2013) as they are more appropriate with small sample sizes. However, they do provide a more conservative estimate of effect. Marquis (2013) conducted further analysis and considered if the child was taught individually or in a sibling pair. It was reported that single children had significant improvements on word reading, sentence comprehension, reading composite and maths, while sibling pairs only indicated significance for math computation. Evaluation found that ADHD, mental health, internalised and externalised behaviours, as defined by the Child Behavior Checklist, moderated the relationship between the intervention and academic skills.

The 25‐week group‐level Teach Your Children Well also assessed academic skills, although the WRAT‐4 has not been validated for use with the aboriginal population, who comprised the majority of the study sample (Harper & Schmidt, 2012). The study found a significant effect on reading (*E.S. = *0.42, *p = *.002) and spelling (*E.S. = *0.38*, p = *.004), but not sentence comprehension or math computation, although the latter fell within the substantively important range. Harper's (2012) evaluation of the 30‐week, group‐level Teach Your Children Well found an effect on reading (*E.S. = *0.40), spelling (*E.S. = *0.25, *p = *.02), and math computation (*E.S. = *0.34, *p = *.044), but not sentence comprehension. The study found a moderating role for school stability on reading scores, with only a significant effect for the intervention when school instability was high (*p < .001*) or medium (*p < .001*). There was also evidence of ADHD as a moderator. Variation in effect across subsets of academic skills between the individual‐level Teach Your Children Well (Flynn *et al*., 2011; 2012; Marquis, 2013) and the group‐level Teach Your Children Well (Harper 2012; Harper & Schmidt, 2012) is explained by differences in the individual and group format and the way components were implemented.

Five studies reporting on four interventions found no evidence of effect. Green *et al*.'s (2014) evaluation of group‐based Multidimensional Treatment Foster Care for Adolescents indicated no impact on scholastic or language skills. Mooney *et al*.'s (2016) evaluation of the Letterbox Club reported no effect on reading accuracy, reading comprehension or reading rate. In the trial of education specialists Zetlin et al. (2004) reported differences between the intervention group and control group at baseline but no significant differences at follow‐up for maths test achievement scores (*p = *.082) or reading test achievement scores (*p = *.448). The ESTEP programme found no impacts on letter word identification, calculation or passage comprehension (Courtney *et al*., 2008; Zinn & Courtney, 2014). The authors hypothesise that a large number of young people enter care due to mental health and behavioural problems, with this being evidenced by the fact that 6.5% of the study sample tested positive for post‐traumatic stress, 35.1% reported as having been in special educational programmes prior to the study, and 26.1% reported a learning disability. They suggest that the graduate students who delivered the intervention did not have the specialist training necessary to serve these youth, and specialist teachers may be more appropriate delivery agents.

#### Academic achievement and grade completion

Three studies reporting on two interventions measured Grade Point Average (GPA), General Education Development (GED) or grade completion (Zetlin *et al*., 2004; Courtney *et al*., 2008; Zinn & Courtney, 2014). Education specialists indicated no impact at 24 months post‐baseline (Zetlin *et al*., 2004). The ESTEP programme found no effect on grade completion at 26.8‐month follow‐up (Courtney *et al*., 2008; Zinn & Courtney, 2014).

#### Special education status

One study, evaluating education specialists, assessed special education status (Zetlin *et al*., 2004). At baseline, 18 young people in the intervention group were in special education, and this was reduced to nine at 24‐month follow up. In the control group the number decreased from 10 to seven. The significance of these findings is not presented.

#### Homework completion

One study reported on homework completion. Multidimensional Treatment Foster Care for young girls leaving the youth justice system was evaluated for homework completion on three days in a one‐week period at 3–6 months and 12 months post‐baseline (Leve & Chamberlain, 2007). At both time points the intervention group spent more days on homework than the control group; individuals in the intervention group spent approximately 150% more days in a week on homework at 12 months post‐baseline, while the control group experienced a decline in the time allocated to this task.

#### School attendance, suspension and drop out

Four studies reporting on four interventions assessed school attendance, with two finding some evidence of effect. Multidimensional Treatment Foster Care for young girls leaving the juvenile system had an effect at the *p *<* *.05 level at 12 months post‐baseline (Leve & Chamberlain, 2007). Fostering Individualized Assistance Programme showed no significant difference in extreme school absences (>40% of school days missed) between the intervention and control group at follow‐up, but when the sample was restricted to the older subset (11.5–16 yrs) the control group was more than two times as likely to be engaged in school absenteeism (Clark *et al*., 1998). Green *et al*.'s (2014) evaluation of the Multidimensional Treatment Foster Care for Adolescents found no effect on attendance (*OR = *2.5, *95% CI = *0.48,13.1). In the evaluation of educational specialists, Zetlin *et al*. (2004) found there was no significant difference between groups at baseline but one at 24‐month follow‐up in favour of the controls.

One study addressed suspension rates. Fostering Individualized Assistance Programme indicated that at 42‐month follow‐up those in the control group were 2.5 times more likely to engage in an extreme proportion of days on suspension (>1% of school days) (Clark *et al*., 1998). When the population was separated into a younger and older subset, there was no significant effect for the younger group but a significant impact was retained for the older category, with the control group being more than four times as likely to be suspended.

Two studies considered school stability and drop‐out. On the Way Home reported that young people in the control group were more than three times more likely to leave school compared to those in the intervention group at 12‐months post‐baseline (*E.S. = *0.30, *95% CI = *0.12,0.75) (Trout *et al*., 2013). However, it is noted that both groups tended to fare better than youth in comparable studies of populations of disabled young people, suggesting that these individuals were better prepared for the transition from out‐of‐home care. Clark *et al*. (1998) also measured school drop‐out and found no significant effects, even when the group was separated into a younger and older subset.

#### Number of school placements

Two evaluations reporting on two interventions measured the number of school placements, with no indication of effectiveness. Zetlin *et al*. (2004) assessed the number of schools attended by young people prior to the introduction of educational specialists. At 24 months post‐baseline the number of schools attended dropped from an average of 1.30 to 1.18 in the intervention group, and from 1.28 to 1.12 in the control group. There was no significant difference between the group at baseline but significant at the *p *<* *.05 level at follow‐up, with suggestion of a more favourable outcome in the control group. Clark *et al*.'s (1998) evaluation of the family‐specialist coordinated programme did not find any impact on the extreme number of school‐to‐school movements, which is defined as more than three placements per year.

#### Teacher‐student relationships

One intervention, Head Start, measured teacher‐student relationships (Lipscomb *et al*., 2013). At six months post‐baseline there was a significant effect for the intervention (*ß = *0.30, *p *<* *.01), with an indirect intervention effect at 18 months.

#### School behaviour

The ESTEP programme assessed impact on school behaviour, which was a composite measure of: getting along with teachers; paying attention in school; getting your homework done; getting along with other students; and arriving on time for class (Courtney *et al*., 2008; Zinn & Courtney, 2014). At approximately 2 years post‐baseline the intervention demonstrated no effect.

#### Academic attitudes

The Letterbox Club measured impact on attitude towards reading, which included recreational reading and academic reading, in addition to liking of school (Mooney *et al.,* 2016). At approximately eight months post‐baseline there was no impact on these outcomes.

### Process evaluation

Process evaluation data was abstracted according to intervention reach, adherence and acceptability to both delivery agents and participants.

Three studies addressed intervention receipt. In the ESTEP programme only 61% of the intervention group received the programme (Courtney *et al*., 2008; Zinn & Courtney, 2014). This was explained by the average length of 15.3 weeks between assignment to the intervention and actual commencement, with 13% waiting between 24 weeks and 2 years to start. Due to the transience of placements, many youth were no longer situated in the foster home listed for tutoring at the time of commencement. The individual‐level Teach Your Children Well reported a number of endogenous and exogenous barriers to uptake, including: busy caregivers; conflict between carer and children; the child was already doing well in school; carer illness; changes in young people's placements; or practical barriers to completing evaluation assessments (Flynn *et al*., 2012). As a result of these factors 29% of the intervention group did not receive tutoring.

Nine studies reporting on five interventions documented adherence, with measurements suggesting variation in implementation practices across studies. Pears *et al*.'s (2013) evaluation of Kids in Transition to School reported high levels of adherence, with 100% of intervention materials being covered in the caregiver group and 98% in the school readiness group. In the ESTEP programme, Courtney *et al*. (2008) found that 28% of young people received less than 20 hours, 33% received between 21 hours and 40 hours, and 28% received more than 40 hours, meaning a number of participants received less than the intended intervention amount. Green *et al*. (2014) monitored dose of Multidimensional Treatment Foster Care for Adolescents, and found that by the end of the intervention only 45% of participants remained in their placement, meaning that the full course of the programme was not delivered to many. In the individual‐level Teach Your Children Well (Flynn *et al*., 2011; 2012; Marquis, 2013), 21 cases reported high fidelity, 2 medium fidelity and 7 low fidelity. Although there was a battery of assessments of delivery, including post‐test questionnaires and weekly performance data, there were challenges in reliably assessing fidelity for the maths curriculum, as the self‐paced, computer based format was looser and more informal than the reading curriculum. The group‐based Teach Your Children Well also reported issues with fidelity to the maths curriculum, where tutors struggled in delivery (Harper, 2012; Harper & Schmidt, 2012). Although Clark *et al*. (1998) did not quantify adherence they offer further insight into how implementation problems may emerge, commenting that adherence may be impacted by variations in delivery agents, the quality and consistency of supervisors for these individuals, and the broader context of social care with high caseloads and transient young people.

Despite inclusion of process evaluation data in studies, there was limited linkage to outcome data or mediator or moderator analysis. Marquis's (2013) evaluation of the individual‐level Teach Your Children Well was the only study to construct implementation as a moderator for intervention outcomes, identifying that higher levels of fidelity in delivering the reading curriculum offered an advantage in maths scores. The same trend was apparent for the maths curriculum, with those receiving a higher level of exposure making significantly higher gains on math computation.

Five studies reporting on three interventions explored acceptability for both delivery agents and participating young people. In the Teach Your Children Well intervention, 79% of foster parents stated they would recommend it, with a further 14% claiming they would recommend it with hesitation (Flynn *et al*., 2011; 2012; Marquis, 2013). The acceptability to the young people was not reported, although there was discussion of challenging behaviour and resistance to tutoring. The ESTEP programme indicated conflict with the large number of additional educational intervention available, with some young people preferring school‐based approaches to those delivered at home, potentially due to them being less stigmatising (Courtney *et al*., 2008). In the evaluation of the Letterbox Club acceptability among children was variable. Some participants expressed appreciation of individual books, while some felt that the included materials were not pitched at the right level or they had received a book as a gift at a different time. One of the key findings was that the intervention's theory of change is based on the hypothesis that children's personal ownership of books will increase their interest and motivation. However, some participants were found to already be ‘book burdened’ rather than ‘book deprived’ and increasing access to such resources did not necessarily increase reading opportunities (Mooney *et al*., 2016).

### Economic evaluation

No studies incorporated a full economic evaluation of the intervention.

### RCT in progress: Fostering healthy futures

Expert recommendation identified an RCT currently being undertaken and due to report imminently. We highlight this study for inclusion in future summaries of research in this area. The Fostering Healthy Futures programme is a manualised skills group that aims to reduce stigma and provide opportunities to learn social and emotional competencies within a supportive environment. (Taussig *et al*., 2007; Taussig & Culhane, 2010; Taussig *et al*., 2012). Groups are delivered for 30 weeks, lasting approximately 1.5 hrs each week, and comprise two trained facilitators and 8–10 children. The intervention is informed by the evidence‐based PATHS curriculum and the Second Step approach. Mentoring is also provided by graduate students in social work, who act as a role model and advocate for the young person, meeting with them for 2–4 hours per week. An RCT has been conducted with 156 children aged 9–11 in foster care due to maltreatment. The primary outcome was mental health, and at 15 months post‐baseline the intervention groups scored significantly lower on multi‐informant measures of poor mental health (*RR = *−0.51, *95% CI = *−0.84,−0.19). Secondary educational outcomes were measured as part of the trial and analysis is currently being undertaken.

## Discussion

The present systematic review has sought to ascertain the effectiveness of educational interventions for LACYP. Fifteen studies reporting on 12 interventions were retrieved. Study designs comprised RCTs, which should offer the most scientifically robust evidence. Of these interventions, nine suggested impact on a range of educational outcomes. Five interventions reported an effect for academic skills: Kids in Transition (Pears *et al*., 2013); Headstart (Lipscomb *et al*., 2013); the individual‐level Teach Your Children Well (Flynn *et al*., 2011; 2012; Marquis, 2013); and both the 25‐week and 30‐week group‐based Teach Your Children Well (Harper, 2012; Harper & Schmidt, 2012). One intervention reported an effect for homework completion: Multidimensional Treatment Foster Care for girls leaving the youth justice system (Leve & Chamberlain, 2007). Three interventions reported an effect for school attendance, suspension or drop‐out: Multidimensional Treatment Foster Care (Leve & Chamberlain, 2007); Fostering Individualized Assistance Programme, which had an impact on extreme absences and suspension, although these effects were largely found for the older subset only (Clark *et al*., 1998); and On the Way Home (Trout *et al*., 2013). One intervention reported an effect for teacher‐student relationships: Head Start (Lipscomb *et al*., 2013). The impact on number of school placements is unclear. No interventions demonstrated an improvement on academic achievement and grade completion; school behaviour; or academic attitudes. Green *et al*.'s (2014) Multidimensional Treatment Foster Care for Adolescents, the Letterbox Club (Mooney *et al*., 2016), and the ESTEP programme (Courtney *et al*., 2008; Zinn & Courtney, 2014) found no indication of effect for any outcome measured.

While some studies indicated methodological rigour, there was extensive variation in conduct and reporting. As a result, no definitive statements should be made with regards to effect, and the aforementioned outcomes should be treated with caution. Indeed, with the exception of the individual‐level Teach Your Children Well (Flynn *et al*., 2011; 2012; Marquis, 2013) and the 30‐week group‐level Teach Your Children Well (Harper, 2012), evaluations did not have large enough sample sizes to detect the anticipated effect size or did not report a power calculation. The inadequacy of reporting also ensured that a number of the risk of biases, as assessed by the Cochrane collaboration tool, were unclear. A number of studies defined themselves as RCTs simply by merit of the fact that a sample was randomly allocated into an intervention or control group. Where bias was identified, pertinent issues included: lack of trial protocols in order to assess selective outcome reporting; incomplete data with unequal attrition across groups; contamination; and inadequate control for key individual, family and social‐level covariates thorough multi‐variate analysis.

The issue of contamination offers insight into the complexity of conducting RCTs within social contexts where notions of ‘usual care’ can be amorphous and the transience of populations can make it problematic to retain the integrity of randomisation. Definitions of ‘usual care’ were limited in studies, and we can only presume a high degree of international variation between child welfare and educational systems, meaning that intervention effects may be underestimated in some instances where usual care is more comprehensive. For example, in the evaluation of ESTEP programme, not only did 12.3% of the control group receive the intervention, but almost 18.9% received school‐based tutoring from a different provider, which undermined intention to treat analysis and potentially reduced the intervention effect (Courtney *et al*., 2008; Zinn & Courtney, 2014). Elsewhere is has been noted that involvement in trials can put a strain on the control group, especially where providers can become more innovative as exposure to knowledge about new approaches causes them to reflect on their own practices, meaning ‘usual care’ evolves over time (Hawe *et al*., 2015).

Process evaluation data revealed some issues with intervention delivery, which may have importance for study replication, while also introducing the risk of Type 3 errors, whereby interventions are rejected for being inherently theoretically faulty when the problem lies with implementation procedures. On balance, interventions were delivered with high fidelity (Flynn *et al*., 2011; 2012; Marquis, 2013; Pears *et al*., 2013), although problems with receipt were routinely encountered. These issues need foregrounding more clearly in order to inform understanding of both the feasibility and acceptability of various interventions, and the utilisation of pilot trials prior to a full‐scale evaluation would provide scope to resolve some of these problems.

There is also a continued need to provide detailed accounts of the contextual specificities within which interventions are delivered and how they impact upon programme theory, especially given child welfare and educational systems may vastly differ both within and across nations. Indeed, the influence of cultural contexts may limit the replication of intervention effects in other settings, while raising questions about the extent of adaptation required. The review indicated that evaluations were conducted in a limited range of countries, with all but two being undertaken in North America. Equally, Teach Your Children Well was the only intervention to be replicated, although this was in the same context as the original study (Flynn *et al*., 2011; Flynn *et al.,* 2012; Harper, 2012; Harper & Schmidt, 2012; Marquis, 2013). Thus the external validity of existing intervention outcomes remains unclear.

A further key omission from studies was economic evaluation conducting a cost‐benefit analysis of the relative savings offered in comparison to usual care. Within a context where the efficient and accountable utilisation of public resources it increasingly important, it is imperative to understand the economic advantages of new interventions. As organisational theory posits (Rogers, 2003), cost savings is a key criterion against which new interventions are assessed for adoption, suggesting that evidence of effect is important but not sufficient.

### An opportunity for scientifically robust evaluation of educational interventions

The utilisation of RCTs within educational and social care settings have been plagued by a wealth of problems that have arguably contributed to their poor conduct, with this issue being further compounded by the declining quality of reporting (Torgerson *et al*., 2005; Flynn *et al*., 2012; Dixon *et al*., 2014; Mezey *et al*., 2015). As Dixon *et al*. (2014, p. 1564) surmise, controversy over trials are derived from the interpretation that they are ‘unethical, positivist, uncritically imported from other disciplines, and unable to yield the certainty they promise’. Indeed, debates have abounded about the perceived unequitable or unethical assignment of individuals to control groups (Gueron, 2008; Dixon *et al*., 2014; Mezey *et al*., 2015), as though the expenditure of public resources on interventions without evidence of effectiveness does not pose its own ethical challenges. Further, more pragmatic limitations have included insufficient recruitment to ensure statistical power, practitioners circumventing randomisation processes by the introduction of their own rationing strategies, and the struggle to secure funding for the conduct of the trial itself (Gueron, 2008; Dixon *et al*., 2014; Mezey *et al*., 2015). However, regardless of these challenges, RCTs within educational and social care settings are increasingly gaining traction, and the research community has openly called for a stronger culture around this evaluation design (Gueron, 2008). While we might welcome this paradigmatic shift, where trials are routinely employed in the evaluation of complex social interventions, it is important to remain critically engaged in their advancement and continually illuminate and debate opportunities to strengthen their conduct and reporting.

In the first instance, we need to move beyond an understanding of theoretically driven interventions as the highly manualised and routinised approaches that are generally perceived as being featured in trial evaluations. Rather, complex social interventions are increasingly conceived as ‘events in systems’, and the work of Penny Hawe has been instrumental in defining programmes by their standardised functions as opposed to composition (Hawe *et al*., 2004). Thus, when interventions are replicated across settings, we should expect them to manifest in different ways in order to accommodate contextual specificities, while activating the same set of causal processes in the generation of outcomes. Logic models, which depict the causal pathway from inputs to outcomes, are important tools in allowing us to define this more complex understanding of intervention theory (Moore *et al*., 2014). As an extension of this theoretical work, there have also been recent calls for the introduction of dark logic models, which consider the potential harmful impacts of interventions (Bonell *et al*., 2015). Indeed, a number of unintended consequences have been identified in complex social interventions for vulnerable populations due to the negative experiences of targeting, but these are often elided in programme development and evaluation.

Second, in response to evolving understandings of complex interventions, we need to ensure that RCTs have the capacity to meet the demands of evaluation. This may involve meaningful engagement with critiques that RCTs fail to capture the intricacies and nuances of our social world (see discussion by Dixon *et al*., 2014), by continually reiterating the sophistication of this study design compared to the reductionist, positivist assumptions often attributed to it. An emerging body of work derived from critical realism (Bhaskar, 1978), and embodied within realist RCTs, has been at the forefront of dispelling many of these presumptions (Bonell *et al*., 2012). As Bonell *et al*. (2012) maintain, trials do not overlook or bracket out complexity but rather embrace it. They fully take into account social causation because the only systematic difference between the intervention and control group is the intervention, and the influence of individuals’ social worlds remain largely undisturbed by the research process. Equally, within this approach, the features of different settings are privileged from the outset due to an emphasis on CMO configurations, where evaluation focuses on understanding how *context* interacts with the intervention's causal *mechanisms* in the generation of *outcomes* (Pawson & Tilley, 1997). The proliferation of implementation science, which offers a number of theoretical frames for exploring how intervention delivery may compromise effectiveness, offers further nuance in the interpretation of the outcome data presented by RCTs (Evans *et al.,* 2015).

Third, there are a number of frameworks, theories and mnemonics within public health that can support effectiveness evaluations within education and social care. The Medical Research Council's *Framework for the Development and Evaluation of Complex Interventions* [Medical Research Council (MRC), 2000; Craig *et al*., 2008] has much to offer in the generation of theoretically informed effective interventions. The sequential investigative phases include: development of an intervention's theoretical rationale through consultation of relevant literature and stakeholders; modelling of processes and outcomes to identify underpinning ‘active ingredients’; an exploratory trial to assess acceptability, recruitment and retention, and to undertake sample size calculations; a full scale effectiveness trial, which also addresses cost effectiveness; and a translational phase to ensure scale‐up and routinisation. Within the conduct of these phases, it may be of value to consult risk of bias checklists, such as those issued by Cochrane, to anticipate and mitigate against the various sources of bias that may emerge. Recently issued process evaluation guidance by the MRC, which explores the generation of logic models and the assessment of implementation, may also offer support in evaluation (Moore *et al*., 2014).

Fourth, there remains a need to improve the reporting of RCTs across educational and social care settings. As illustrated in the present systematic review, and highlighted by Torgerson *et al*. (2005), quality has been extremely poor and it is difficult to differentiate between studies that are scientifically weak with a lack of internal or external validity, and those that have simply been reported inadequately. There are a number of processes and procedures for enhancing reporting. Trial protocols should be published detailing the primary and secondary outcome measures to be evaluated. This is important for assessing the completeness of outcome data at the individual trial level, but also for understanding the landscape of effective interventions and the extent of publication bias. Reporting of outcome data should adhere to standardised statements, such as CONSORT (Consolidated Standards of Reporting Trials). Although there remains debate about the adequacy of CONSORT, current extensions are in development to sensitise the statement to social and psychological interventions under the rubric of CONSORT‐SPI (Montgomery *et al*., 2013).

### Limitations

The limitations of the studies included in this review have been largely addressed through risk of bias assessments, and further reflections on the conduct and reporting of RCTs within education and social‐care settings. Studies are further limited by a lack of external validity, with only two evaluations being conducted outside North America. Limitations also pertain to the review methodology. First, although a sensitive and comprehensive strategy was undertaken, consultation with experts in the field revealed that effectiveness evaluations are predominantly reported within the grey literature, largely in the form of unpublished theses or government reports (Forsman & Vinnerljung, 2012). Subsequently, a number of evaluations may not have been detected. Yet as discussed, RCTs are increasingly being utilised, with Fostering Healthy Futures due to report imminently, and in light of this rapid progress we would suggest the repetition of this review in the near future. Second, the heterogeneity of studies ensured the review is limited by the presentation of a narrative synthesis as opposed to meta‐analysis. Third, the inclusion of RCTs ensures that a number of forms of ‘evidence’ have not been included. However, the review aimed to assess methodologically the scientific robustness of evaluation, and restriction to this design permitted full consideration of this objective.

## Conclusion

The present review has assessed the effectiveness of educational interventions targeted to LACYP. Based on the included studies it is premature to make any claims about intervention impact due to the variable quality of study conduct and reporting. Thus, although we might encourage the continued utilisation of RCTs in generating evidence, this progress needs to be accompanied with more critical monitoring of methodological quality. Evaluation and reporting guidance, such as the Medical Research's *Framework for the Development and Evaluation of Complex Interventions* and CONSORT‐SPI may be employed to strengthen the study design, and it is only with the introduction of such rigour and robustness that we can start to draw conclusions around what works, for whom and in what contexts.
